# Design and Uncertainty Analysis for a PVTt Gas Flow Standard

**DOI:** 10.6028/jres.108.004

**Published:** 2003-02-01

**Authors:** John D. Wright, Aaron N. Johnson, Michael R. Moldover

**Affiliations:** National Institute of Standards and Technology, Gaithersburg, MD 20899-0001

**Keywords:** correlated uncertainty, gas flow standard, inventory volume, mass cancellation, *PVTt* standard, sensor response, uncertainty

## Abstract

A new pressure, volume, temperature, and, time (*PVTt*) primary gas flow standard at the National Institute of Standards and Technology has an expanded uncertainty (*k* = 2) of between 0.02 % and 0.05 %. The standard spans the flow range of 1 L/min to 2000 L/min using two collection tanks and two diverter valve systems. The standard measures flow by collecting gas in a tank of known volume during a measured time interval. We describe the significant and novel features of the standard and analyze its uncertainty. The gas collection tanks have a small diameter and are immersed in a uniform, stable, thermostatted water bath. The collected gas achieves thermal equilibrium rapidly and the uncertainty of the average gas temperature is only 7 mK (22 × 10^−6^ *T*). A novel operating method leads to essentially zero mass change in and very low uncertainty contributions from the inventory volume. Gravimetric and volume expansion techniques were used to determine the tank and the inventory volumes. Gravimetric determinations of collection tank volume made with nitrogen and argon agree with a standard deviation of 16 × 10^−6^ *V_T_*. The largest source of uncertainty in the flow measurement is drift of the pressure sensor over time, which contributes relative standard uncertainty of 60 × 10^−6^ to the determinations of the volumes of the collection tanks and to the flow measurements. Throughout the range 3 L/min to 110 L/min, flows were measured independently using the 34 L and the 677 L collection systems, and the two systems agreed within a relative difference of 150 × 10^−6^. Double diversions were used to evaluate the 677 L system over a range of 300 L/min to 1600 L/min, and the relative differences between single and double diversions were less than 75 × 10^−6^.

## 1. General Description of a *PVTt* Gas Flow Standard

**Fig. 1:**
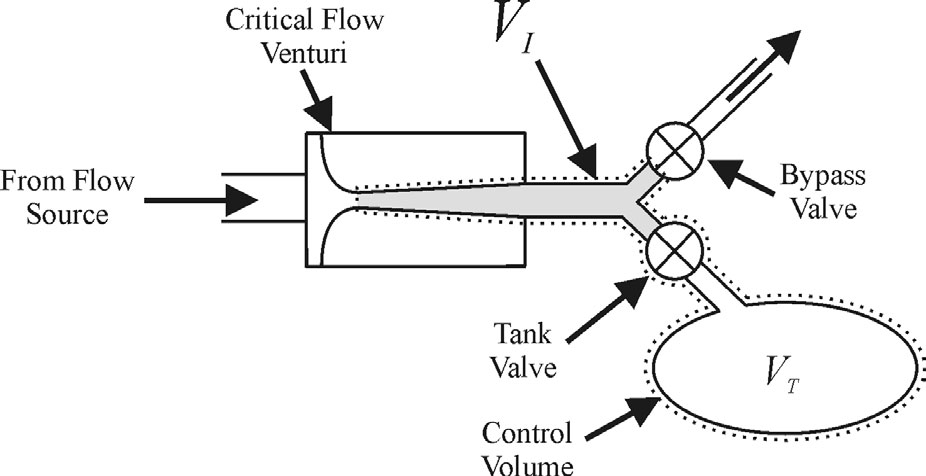
Arrangement of equipment in a *PVTt* system.

*PVTt* systems have been used as primary gas flow standards by the National Institute of Standards and Technology (NIST) and other laboratories for more than 30 years [1,2,3,4]. The *PVTt* systems at NIST consist of a flow source, valves for diverting the flow, a collection tank, a vacuum pump, pressure and temperature sensors, and a critical flow venturi (CFV) which isolates the meter under test from the pressure variations in the downstream piping and tank (see Fig. 1).

The process of making a *PVTt* flow measurement normally entails the following steps:

1. Close the tank valve, open the bypass valve, and establish a stable flow through the CFV.
2. Evacuate the collection tank volume (*V_T_*) with the vacuum pump.
3. Wait for pressure and temperature conditions in the tank to stabilize and then acquire initial values for the tank $\left(P_{T}^{i}\;\text{and}\;T_{T}^{i}\right)$. These values will be used to calculate the initial density and the initial mass of gas in the tank $\left(m_{T}^{i}\right)$.
4. Close the bypass valve and during the “dead-end time” when both the bypass and tank valves are fully closed, obtain a start time (*t* ^i^). At the same time, acquire the initial pressure and temperature in the inventory volume $\left(P_{I}^{i}\;\text{and}\;T_{I}^{i}\right)$. These values will be used along with the equation of state for the gas and the inventory volume (*V*_I_) to obtain an initial mass in the inventory volume $\left(m_{I}^{i}\right)$. Shortly after the bypass valve is fully closed, open the tank valve.
5. Wait for the tank to fill to a prescribed upper pressure (in this system, about 100 kPa), and then close the tank valve and obtain the stop time (*t*^f^) during the dead-end time. At the same time, acquire the pressure and temperature in the inventory volume $\left(P_{I}^{f}\text{and}\;T_{I}^{f}\right)$ and hence the final mass in the inventory. Open the bypass valve.
6. Wait for stability and then acquire $P_{T}^{f}$ and $T_{T}^{f}$ and hence $m_{T}^{f}$.

By writing a mass balance for the control volume composed of the inventory and tank volumes (see the volume defined by the dashed line in Fig. 1), one can derive an equation for the average mass flow during the collection time:

$$\dot{m}=\frac{\left(m_{T}^{f}-m_{T}^{i})+(m_{I}^{f}-m_{I}^{i}\right)}{t^{f}-t^{i}},$$ (1)

or, neglecting the volume changes between the initial and final conditions:

$$\dot{m}=\frac{V_{T}(\rho_{T}^{f}-\rho_{T}^{i})+V_{I}(\rho_{I}^{f}-\rho_{I}^{i})}{t^{f}-t^{i}},$$ (2)

where *ρ* is the gas density determined via a real gas equation of state:

$$p=\frac{PM}{ZRT},$$ (3)

where *Z* is the compressibility factor, *M* is the molecular weight, and *R* is the universal gas constant.

The start and stop times can be chosen at any point during the dead-end time as long as the inventory conditions are measured coincidentally. Why is this true? Implicit in the *PVTt* basis equation [Eq. (2)] are two requirements: 1) the measurement of the initial and final densities must be coincident with the measurement of the start and stop times and 2) there must not be any other sources or sinks of mass flow to the control volume. The second condition is met for the entire time that the bypass valve is fully closed, including the start and stop dead-end times. It is not necessary that the initial and final determinations of the mass in the collection tank be done coincidentally with the start and stop times because the tank is free of leaks and it is advantageous to measure these mass values when the tank conditions have reached equilibrium. The freedom to choose the start and stop times from within the dead-end time intervals allows one to choose times where the initial and final inventory densities match, giving essentially zero mass change in the inventory volume (Δ*m_I_*) and extremely good cancellation of certain correlated inventory uncertainties.

Equations (2) and (3) are *basis equations* for the mass flow calculation in a *PVTt* system and hence are the foundation for the propagation of uncertainties analysis [5] which will be presented in later sections. As will be shown, the most important contributors to the uncertainty of the gas flow standard are the collection tank volume, the density of the gas in the full collection tank (both primarily traceable to pressure uncertainty), and uncertainties related to the measurement of the change of mass in the inventory volume.

We use the volumetric flow unit of L/min throughout this publication since the *PVTt* system is a volumetric (as opposed to gravimetric) gas flow standard. The L/min flow unit reflects the fact that the *PVTt* standard works over the same range of volumetric flow regardless of the molecular weight of the gas being metered. We have used the reference conditions of 293.15 K and 101.325 kPa when calculating these volumetric flows, hence the flow unit corresponds to the widely used “standard liter per minute” or slm.

## 2. Design and Operation of the *PVTt* Standard

Experience with a previous *PVTt* flow standard at NIST indicated that improvements in temperature and pressure instrumentation as well as in the design and operation of the new system would be necessary in order to achieve the uncertainty goal of 0.05 % or better. We now describe aspects of the design and operation of the new flow standard that are important to achieve this low uncertainty. Section 2.1 describes the measurement of the average temperature of the collected gas, Sec. 2.2 describes the procedures that minimize the uncertainty of the mass change in the inventory volume, and Sec. 2.3 describes the determination of the tank and inventory volumes.

### 2.1 Average Temperature of the Collected Gas

One of the most important sources of uncertainty in a *PVTt* flow standard is the measurement of the average temperature of the gas in the collection tank, particularly after filling. The evacuation and filling processes lead to cooling and heating of the gas within the volume due to flow work and kinetic energy phenomena [6]. The magnitude of the effect depends on the flow; however, the temperature rise in an adiabatic tank can be 10 K or more. Hence, immediately after filling and evacuation, significant thermal gradients exist within the collected gas. For a large tank, the equilibration time for the gas temperature can be many hours. If the exterior of the tank has non-isothermal or time varying temperature conditions, stratification and non-uniform gas temperatures will persist even after many hours.

**Fig. 2:**
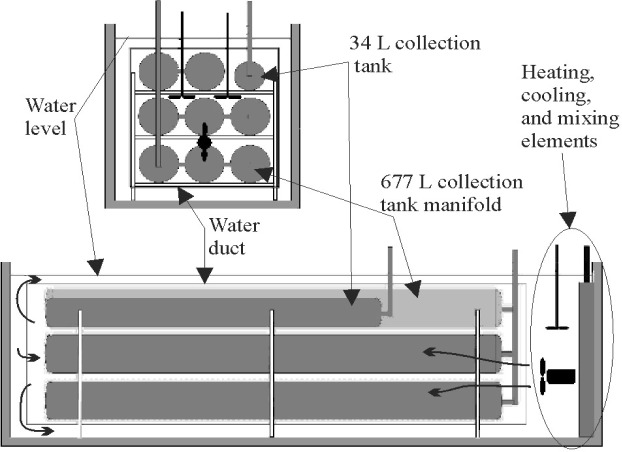
A schematic of the *PVTt* collection tanks, water bath, duct, and temperature control elements.

**Fig. 3:**
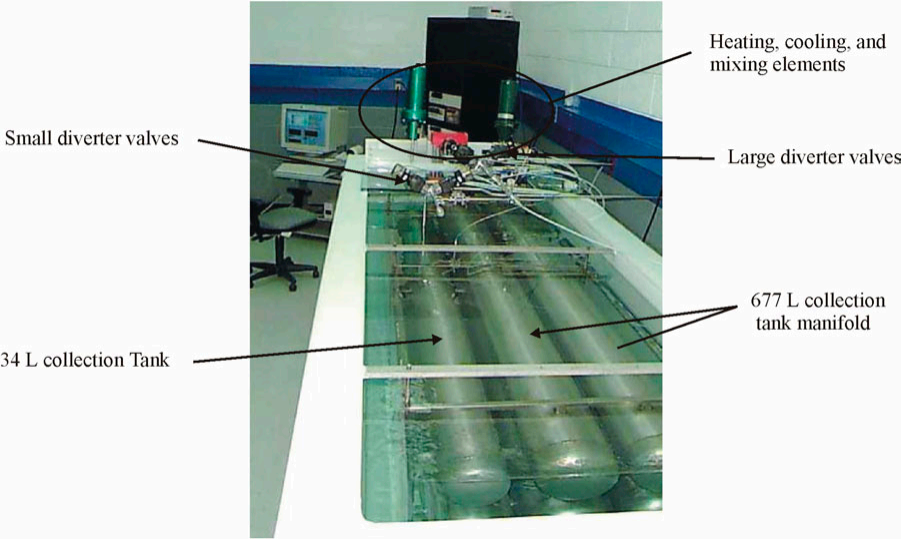
A photograph of the two *PVTt* collection tanks submerged in the temperature controlled water bath.

In this work, we avoided long equilibration times and the difficult problem of measuring the average temperature of a non-uniform gas by designing the collection tanks for rapid equilibration of the collected gas and by immersing the tanks in a well-mixed, thermostatted, water bath (see Fig. 2 and Fig. 3). There are two control volumes, a 34 L collection tank and a 677 L collection tank. Because the equilibration of the 677 L tank is slower, we consider it here. The 677 L tank is composed of eight, cylindrical, 2.5 m long, stainless steel shells connected in parallel by a manifold. Each shell has a wall thickness of *l* = 0.6 cm and an internal radius of *a* = 10 cm. Because all of the collected gas is within 10 cm of a nearly isothermal shell, the gas temperature quickly equilibrates with that of the bath. After the collected gas equilibrates with the bath, the gas temperature is determined by comparatively simple measurements of the temperature of the recirculating water. Remarkably, the water temperature measurements made with 14 sensors had a standard deviation of only 0.4 mK during a typical, 20 minute long, equilibration interval. In Sec. 2.1.1, we describe the bath; in Sec. 2.1.2, we discuss the equilibration of the collected gas.

#### 2.1.1 The Water Bath

The water bath is a rectangular trough 3.3 m long, 1 m wide, and 1 m high. Metal frames immersed in the tank support all the cylindrical shells and a long duct formed by four polycarbonate sheets. The duct surrounds the top, bottom, and sides of the shells: however, both ends of the duct are unobstructed. At the upstream end of the bath, the water is vigorously stirred and its temperature is controlled near the temperature of the room (296.5 K) using controlled electrical heaters and tubing cooled by externally refrigerated, circulated water. A propeller pushes the vigorously stirred water through the duct along the collection tanks. When the flowing water reaches the downstream (unstirred) end of the trough, it flows to the outsides of the duct and returns to the stirred volume through the unobstructed, 10 cm thick, water-filled spaces between the duct and the sides, the top, and the bottom of the rectangular tank.

**Fig. 4:**
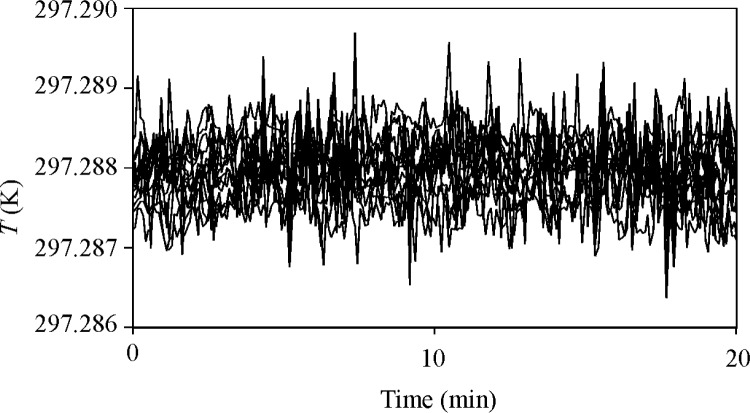
Temperature data for 14 thermistors distributed in the water bath.

The uniformity and stability of the water temperature was studied using 14 thermistors. The thermistors were bundled together and zeroed at one location in the water bath. Then, they were distributed throughout the water bath. Figure 4 plots data recorded at 5 s intervals from these 14 thermistors. Nearly all of the data in Fig. 4 is within ± 1 mK of their mean and the standard deviation of the data from their mean is only 0.4 mK. The largest temperature transients occur where the mixed water enters the duct, indicating incomplete mixing. The tank walls attenuate these thermal transients before reaching the collected gas. Thus, after equilibration, the non-uniformity of the water bath and the fluctuations of the average gas temperature are less than ± 1 mK (3 × 10^−6^ *T*).

#### 2.1.2 Equilibration of the Collected Gas

For design purposes, we estimated the time constant (*τ*_gas_) that characterizes the equilibration of the gas within the collection tank after the filling process. The estimate considers heat conduction in an infinitely long, isotropic, “solid” cylinder of radius *a* [7]. For the slowest, radially symmetric heat mode, *τ*_gas_ = (*a*/2.405)^2^/*D*_T_, where *D*_T_ is the thermal diffusivity of the gas. This estimate gives *τ*_gas_ = 80 s for nitrogen in the 677 L tank. This estimate for *τ*_gas_ is too large insofar as it neglects convection, conduction through the ends of the tanks, and the faster thermal modes, all of which hasten equilibration. The time constants for heat to flow from the gas through the tank walls and the time constant for a hot or cold spot within a wall to decay have been calculated and found to be less than a second. Therefore, we expect the collected gas to equilibrate with a time constant of 80 s or less.

**Fig. 5:**
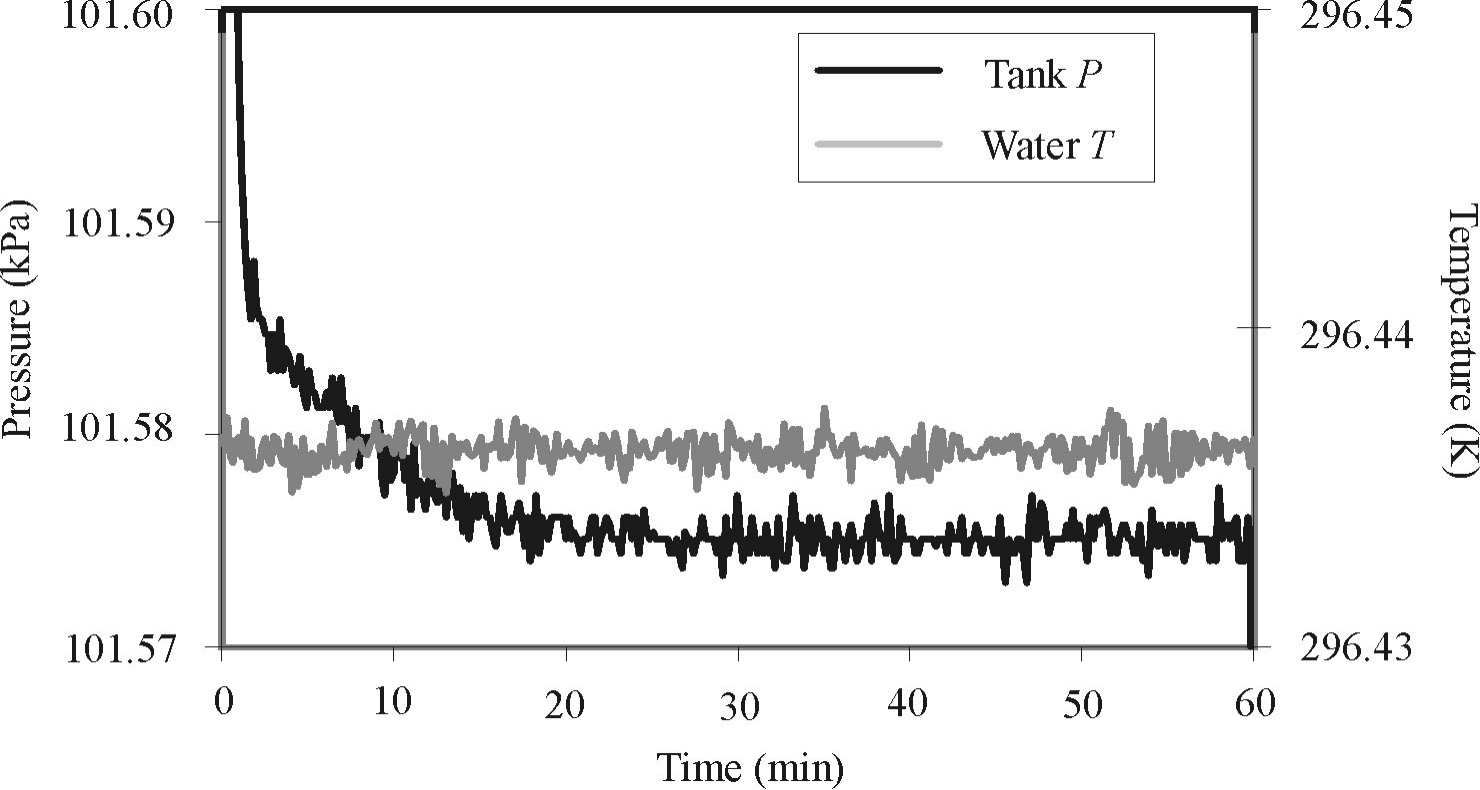
The equilibration of pressure and temperature immediately following a collection tank filling, 25 L/min in the 34 L tank.

The equilibration of the collected gas was observed experimentally by using the tank as a constant-volume gas thermometer. After the tank valve was closed, the pressure of the collected gas was monitored, as shown in Fig. 5. Our analysis of data such as those in Fig. 5 leads to the experimental values *τ*_gas_ of less than 60 s for both the 677 L and 34 L tanks, in reasonable agreement with the estimates. The measured time constant and Fig. 5 show that a wait of 20 minutes guarantees that the collected gas is in equilibrium with the bath, within the resolution of the measurements.

The manifold linking the eight cylindrical shells is completely immersed in the water bath. Thus, the gas in the manifold quickly equilibrates to the bath temperature as well. However, each collection system has small, unthermostatted, gas filled volumes in the tubes that lead from the collection tanks to the diverter valves, the pressure transducers, etc. In Sec. 6.2.1, we show the possible temperature variations of these small, unthermostatted volumes make very small contributions to the uncertainty of the gas temperature and the flow measurements.

### 2.2 Mass Change in the Inventory Volume

#### 2.2.1 Overview and Strategy

**Fig. 6:**
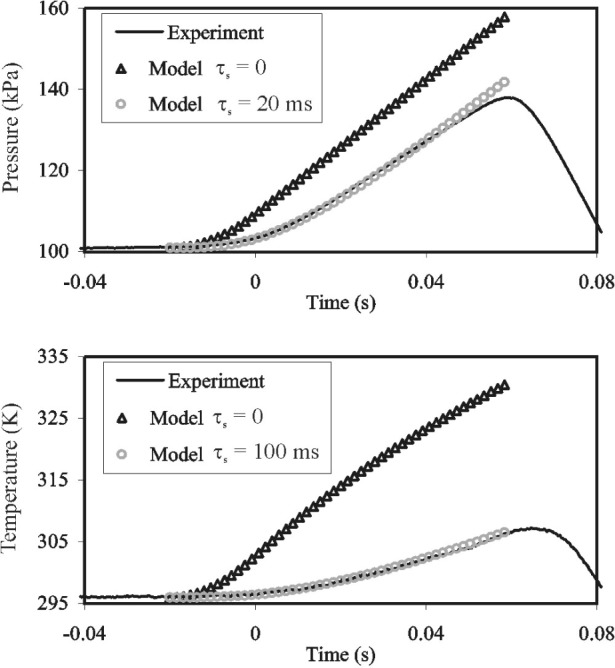
Experimentally measured data (25 L/min in the 34 L tank) and thermodynamic model predictions for zero and non-zero sensor time constants. The model outputs demonstrate that neglect of sensor response causes significant error in the measurement of inventory conditions.

As outlined in Sec. 1, the start time *t* ^i^ and the stop time *t* ^f^ used in Eqs. (1) and (2) are chosen to occur during the brief “dead-end times” (< 100 ms) when both the tank valve and the bypass valve are closed, i.e., we use a “zero overlap” diversion [8]. This choice has the advantage of clear mass balance accountability for all the gas flowing through the critical flow venturi during both diversions and the tank filling. Unfortunately, it is difficult to determine either $m_{I}^{f}$ or $m_{I}^{i}$ and hence the change in mass within the inventory volume accurately (especially at high flows) because both the pressure and the temperature in the inventory volume rise rapidly as the flow through the critical venturi accumulates in the inventory volume (see Fig. 6).

**Fig. 7:**
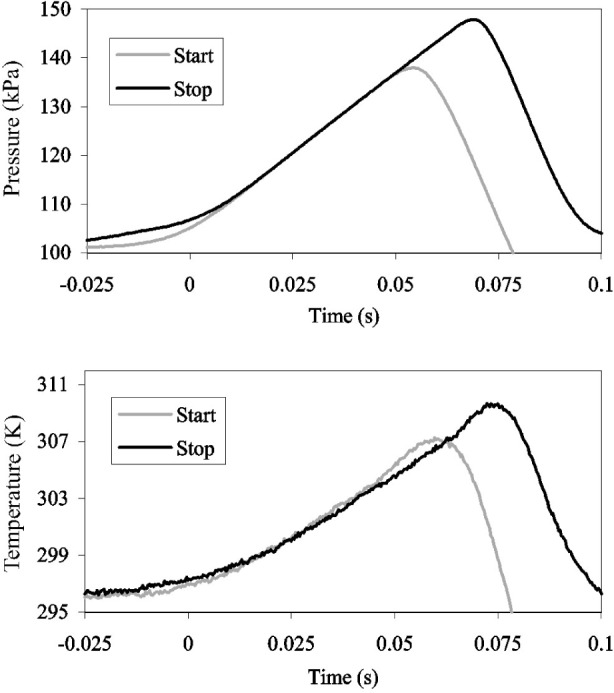
Superimposed inventory data traces for a start diversion and a stop diversion in the 34 L tank at 25 L/min demonstrating “symmetric” diverter valve behavior. The stop dead-end time was approximately 15 ms longer.

Our strategy for dealing with the inventory mass change has two elements. First, by design, the inventory volume *V*_I_ is much smaller than the collection tank volume *V*_T_. (For the 34 L system, *V*_T_/*V*_I_ = 500; for the 677 L system, *V*_T_/*V*_I_ = 700.) Thus, the uncertainty of mass flow is relatively insensitive to uncertainty in $m_{I}^{f}$ and $m_{I}^{i}$ since both are small compared with the total mass of collected gas. Second, we choose *t* ^i^ near the end of the dead-end time and we chose *t*^f^ such that *P*(*t*^i^) = *P*(*t*^f^). These choices define a “mass cancellation” method: since the initial and final inventory densities are essentially equal, Δ*m*_I_ is nearly zero. In fact, we will assume that Δ*m*_I_ is zero and consider the quantity only in terms of flow measurement uncertainty, not as part of the flow calculation. Symmetry of the inventory transients (see Fig. 7) and the mass cancellation method also give uncertainty benefits due to high correlation in the uncertainties of pressure and temperature measurements for Δ*m*_I_.

We tested our strategy for choosing *t*^i^ and *t*^f^ for both the 34 L and the 677 L flow standards. (See Sec. 4 for details of these tests.) To test the 34 L system, we collected identical flows spanning the range 3 L/min to 100 L/min in both the small and the large tanks, using the large tank as a reference for the small tank since its inventory uncertainties are quite small in this flow range. To test the 677 L collection system, we collected identical flows in the 677 L tank following two different protocols. In the first protocol, the inventory volume was dead-ended at the beginning and end of the collection interval in the usual manner. In the second protocol the collection interval was divided into two subintervals, which doubled Δ*m*_I_ and allowed assessment of its uncertainty contribution.

These tests indicate uncertainties due to the inventory volume that are proportional to flow as would be expected based on a thermodynamic model of the inventory pressure and temperature transients. If the inventory uncertainties are considered to arise from uncertainty in the collection time, the inventory mass change uncertainty found experimentally for the 34 L system was $u_{\Delta m_{I}}=4\text{ms}\times \dot{m}(200\times 10^{-6}\dot{m}\;\text{for its maximum flow})$. For the 677 L system, single and double diversions changed the flow measurement by 75 × 10^−6^ $\dot{m}$ or less.

In the remainder of this section, we describe conditions within the inventory volume during the dead-end times using both a model and measurements. The measurements show that *T*(*t*) and *P*(*t*) are nearly the same during the start and stop dead-end times. Finally, we show that Δ*m*_I_ is insensitive to the exact choice of *t* ^i^, provided that the condition *P*(*t*^i^) = *P*(*t*^f^) is applied near the end of the dead-end time.

#### 2.2.2 Conditions Within the Inventory Volume

Figure 6 displays the time dependent temperature *T*(*t*) and pressure *P*(*t*) in the inventory volume of the smaller collection system at a typical collection rate (*m* = 25 L/min; collection time = 82 s). The triangles (*τ* = 0) in Fig. 6 were calculated from the lumped-parameter, thermodynamic model developed by Wright and Johnson [6]. The model assumes a constant mass flow $\dot{m}$ at the entrance to the inventory volume. The model neglects heat transport from the gas to the surrounding structure and non-uniform conditions, such as the jet entering the volume. For Fig. 6, *T*(*t*) and *P*(*t*) were calculated on the assumption that the diverter valve reduced the flow linearly (in time) to zero during the interval −0.02 s < *t* < 0. Experimentally measured values of *T*(*t*) and *P*(*t*) recorded at 3000 Hz (smooth curves) are also shown in Fig. 6. Most of the differences between the measured curve and the (*τ* = 0) calculated triangles result from the time constants of the sensors used to measure *T*(*t*) and *P*(*t*). This is demonstrated by the agreement between the experimental curve and the model results when time constants are incorporated (circles).

In Fig. 6, the calculated curves do not display features that mark either the onset or the completion of the diverter valve closing. Thus, even *T*(*t*) and *P*(*t*) data from perfect sensors cannot be used to mark these events. For this reason, the times *t* ^i^ and *t* ^f^ were chosen at times that were clearly within the dead-end time intervals.

Figure 6 shows that the measured values of *T*(*t*) and *P*(*t*) are consistent with the Wright-Johnson model for the inventory volume after allowance is made for the response times of the sensors. The consistency shows that the behavior of the inventory volume is understood semi-quantitatively. However, this is not sufficient to accurately calculate the density *ρ*(*t*) from measurements of *T*(*t*) and *P*(*t*) because the fraction of the flow collected as the valves are closing cannot be deduced from the measurements. Instead, we relied on the pressure sensor to choose *t*^i^. The pressure sensor is preferred to the temperature sensor because it responds more quickly and also because it responds to the average conditions throughout the inventory volume rather than the conditions at only one location. We choose *t* ^i^ near the end of the dead-end time, where the *P*(*t*) measurements are nearly parallel to the *τ* = 0 model. In this regime, the derivative d*P*/d*t* is large and its dependence on precisely how the valve closed is small. Because the dependence on how the valve closed has decayed, we expect that *P*(*t*) will be the same during the start and the stop dead-end times, improving the mass cancellation as well as the correlation of initial and final inventory density uncertainties.

#### 2.2.3 Near Symmetry of Start and Stop Behavior of *P*(*t*)

Figure 7 shows records of *T*(*t*) and *P*(*t*) taken during the dead-end time intervals at the start and the stop of a single flow measurement. As before, the data were recorded at 3000 Hz for 500 ms and the plots were displaced along the horizontal axis until they nearly overlapped. The pressure and the temperature at the beginning of the start dead-end time were slightly lower than those at the stop dead end time; however, the two records match closely during the dead-end time. This implies that the time-dependent densities *ρ*(*t*) also nearly match.

**Fig. 8:**
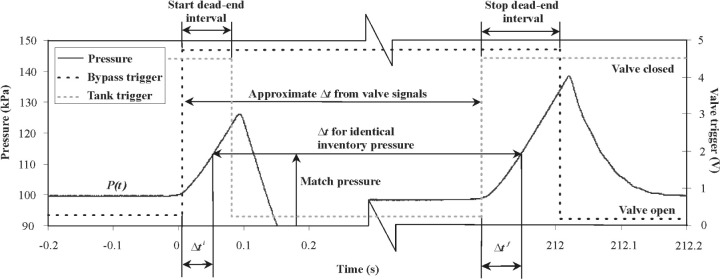
Data records of inventory sensors and valve trigger signals are used to adjust the collection time and improve cancellation of the initial and final inventory mass as well as inventory uncertainties.

At both diversions shown in Fig. 7, valve trigger signals were gathered along with the temperature and pressure measurements using a commercially manufactured data acquisition card (see Fig. 8). The trigger signals originate from an LED/photodiode pair and a flag on the valve actuator positioned so that the circuit output rises to a positive voltage when the valve is closed. These valve signals are used to trigger timers which give the approximate collection time.

As represented in Fig. 8, the inventory record is post-processed by the controlling program to obtain both the initial and final measurements of pressure and temperature in the inventory volume as well as the final collection time. A “match pressure” [*P*(*t*^i^)] is chosen that falls late within the dead-end time. The match pressure value is found in both the start and stop data series and the time differences between the match pressure measurement and the start and stop trigger signals (Δ*t*^i^ and Δ*t*^f^) are determined from the data record. The appropriate time correction is added to the approximate collection time from the timers. Thus, by adjusting the collection time using the inventory data records, the initial and final inventory pressures and temperatures are nearly matched, leading to nearly equal initial and final inventory densities and inventory mass cancellation.

#### 2.2.4 Insensitivity of Δ*m*_I_ to the Match Pressure

**Fig. 9:**
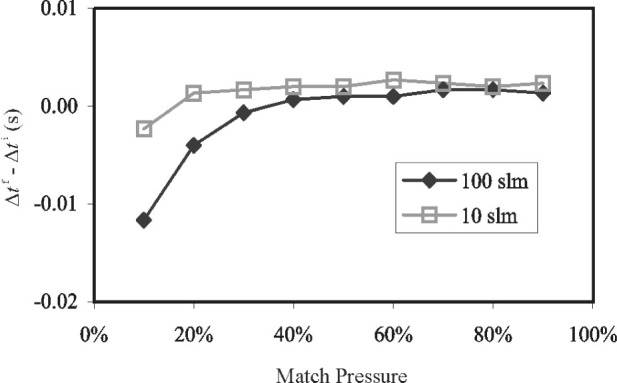
The collection time correction vs the match pressure used in the inventory mass cancellation algorithm.

Figure 9 shows the total correction time as a function of the match pressure for two flows in the 34 L system. The 100 L/min flow is very high for the 34 L tank, having only an 18 s collection time. Match pressure is shown as a percentage of the range of pressures measured during the diversion transient. For a perfectly fast system (valves and sensors), these plots would be horizontal lines, i.e., any chosen match pressure would result in the same time correction. However, for the real system with its inevitable limitations, the match pressure does matter. Exploring the possible reasons for this is valuable for improving the system and for obtaining an accurate uncertainty analysis.

First recall that the inventory sensors have non-zero time constants and therefore the measurements they provide are damped versions of the real conditions and further, the values they report at any given instant are subject to the recent history of the pressure or tempera ture value. Second, realize that perfect symmetry of conditions before and during diversion is unobtainable and that these imperfections and the significance of the sensor damping increase with the flow. For example, at high flows, the rate of change of pressure during the tank filling process is large and it becomes more difficult to make the pressure at which the stop diversion begins closely match the pressure at which the start diversion began (due to sensor response and valve control delays). This “trigger pressure difference” will be considered again in Sec. 4. As another example, the bypass and tank valves may not close at the same speed.

Analysis of the thermodynamic model of the inventory and its sensors shows that times later in the dead-end time give better mass cancellation under these circumstances since the sensor output enters a period with nearly constant slope that is equal to the real pressure slope. The experimental results given in Fig. 9 support this assertion: match pressures between 50 % and 90 % result in nearly constant correction times, while low match pressures (early in the dead-end time) give much larger corrections. Based on this analysis, a match pressure of 80 % has been selected for use in the flow standard. Figure 9 demonstrates the insensitivity of Δ*m*_I_ to a wide range of match pressure values.

Figure 9 also illustrates the concept that uncertainties related to the inventory volume can be treated not only as mass measurement uncertainties, but as time measurement uncertainties as well. One can consider the uncertainty in the measurement of time between conditions of perfect mass cancellation, or one can consider the uncertainty in the measurement of inventory mass differences between the start and stop times. Both perspectives offer insight and verification of the uncertainties of the inventory volume and flow diversion process.

### 2.3 Measurement of the Tank and Inventory Volumes

#### 2.3.1 Gas Gravimetric Method

The volume of the 677 L tank was determined by a gas gravimetric method. In this method, the mass of an aluminum high pressure cylinder was measured before and after discharging its gas into the evacuated collection tank. The change in mass of the high pressure cylinder and the change in density of the gas in the collection tank were used to calculate the collection tank volume. Nominally,

$$V_{\text{grav}}=\frac{m_{c}^{i}-m_{c}^{f}}{\rho_{T}^{f}-\rho_{T}^{i}}-V_{\text{extra}},$$ (4)

where the *m*_c_ indicates the mass of the high pressure cylinder and *V*_extra_ represents the extra volume temporarily connected to the tank for the purpose of introducing the gas from the cylinder to the tank (usually a small volume of tubing and a valve body). The extra volume is calculated from dimensional measurements or measured by liquid volume transfer methods.

In practice, a more complex formula than Eq. (4) was used to account for a small amount of gas that enters the control volume from the room when the cylinder is disconnected from the collection tank since the final tank pressure was less than atmospheric. For the volume determinations performed for the 677 L tank, the effect amounts to only 5 × 10^−6^ *V*_T_.

The volume determination was conducted with both nitrogen and argon gas. In both cases high purity gas was used (99.999 %) and care was taken to evacuate and purge the system to minimize composition uncertainties. When nitrogen was used, the aluminum cylinder weighed approximately 4200 g when filled at 12.5, and approximately 3800 g after it was emptied to 55 kPa. When argon was used, the initial and final masses were 4440 g and 3820 g, respectively. The standard deviation of the six volume measurements (four with nitrogen, two with argon) was 16 × 10^−6^ *V*_T_.

The initial and final masses of the gas cylinder were measured using a substitution process with reference masses and a mass comparator enclosed in a wind screening box. The comparator has a full scale of 10 kg and resolution of 1 mg. The cylinder and a set of reference masses of nearly the same weight were alternated on the scale five times. The zero corrected scale readings were then calibrated to the reference masses and buoyancy corrected via the following formula:

$$m_{c}=\frac{S_{c}}{S_{\text{ref}}}m_{\text{ref}}\left(1-\frac{\rho_{\text{air}}}{\rho_{\text{ref}}}\right)+\rho_{\text{air}}V_{\text{ext}}$$ (5)

where *S* represents the scale reading, ref indicates the reference masses, *ρ*_air_ is the ambient air density where the measurements were conducted, and *V*_ext_ is the external volume of the high pressure cylinder and its valve and fittings. The density of the ambient air was calculated from the barometric pressure, the temperature and humidity inside the wind screen, and an air density formula that includes humidity [9].

The external volume of the high pressure cylinder appears in Eq. (5) due to air buoyancy corrections. The external volume of the cylinder was measured by Archimedes principle, i.e. by measuring the change in apparent mass of the object in two media with differing and known densities. One of the media was distilled water, and the cylinder apparent mass in the water was measured as follows. Liquid was added to the cylinder interior until it was nearly neutrally buoyant in the tank of distilled water. The addition of liquid inside the cylinder has no effect on its external volume. The temperature of the distilled water was raised or lowered (thereby changing the density of the distilled water) until the cylinder was essentially neutrally buoyant. At this point, the apparent mass in the distilled water is zero. The temperature of the distilled water was recorded and its density was calculated via an equation from the literature [10]. Hence, the temperature of the distilled water was used in place of a weigh scale to measure the apparent mass in water. The apparent mass of the cylinder in air (with the liquid still inside) was measured using the comparator described above. The density of air with humidity was calculated as previously described. The external volume of the cylinder was calculated for the nominal room temperature (*T*_ref_) of 296.5 K with the following formula:

Vext(Tref)=mairA−mwaterA[ρwater[1+3α(Twater−Tref)]−ρair[1+3α(Tair−Tref)]], (6)

where the superscript A indicates apparent mass and *α* is the coefficient of thermal expansion for the aluminum tank. The terms containing *α* correct for changes in the cylinder volume due to differences between the water temperature, the air temperature, and the reference temperature. However, for this particular case, these thermal expansion issues could have been neglected since both the water temperature and air temperature never differed from *T*_ref_ by more than 1.5 K. The thermal expansion corrections to the external volume were less than 0.5 cm^3^ or 100 × 10^−6^ *V*_ext_ and the external volume has a small sensitivity coefficient in the collection tank volume determination process.

The expansion of the external cylinder volume as a function of its internal pressure was not negligible. The Archimedes principle measurements showed a volume increase from 4697.5 cm^3^ to 4709 cm^3^ between the 100 and 12.5 Mpa pressures. This change agreed well with predictions based on material properties, and the appropriate experimental values for external volume were used in the cylinder mass calculations [Eq. (5)], depending on whether the cylinder was empty or full. If this issue were neglected, it would lead to relative errors in the mass change measurements of about 35 × 10^−6^.

#### 2.3.2 Volume Expansion Method

The 34 L collection tank volume, the inventory volume for the large collection tank, and the small inventory volume were all determined with a volume expansion method. In this method, a known volume is pressurized, the unknown volume is evacuated, a valve is opened between the two volumes, and the density changes within the two volumes are used to calculate the unknown volume. Applying conservation of mass to the system of the two tanks yields:

$$V_{2}=\frac{(\rho_{1}^{f}-\rho_{1}^{i})V_{1}}{\left(\rho_{2}^{i}-\rho_{2}^{f}\right)}-V_{\text{extra}},$$ (7)

where the subscripts 1 and 2 refer to the known and unknown volumes, respectively. As before, the density values are based on pressure and temperature measurements of the gas within the volumes and gas purity issues must be considered. Note that in many cases the final densities can be considered the same in both volumes 1 and 2, but for the determination of the 34 L tank volume, elevation differences between the two tanks required a head correction to the pressure measurements and therefore the two densities were not strictly equal. The difference in elevation resulted in a relative difference in gas density of 20 × 10^−6^ even though the two tanks were connected.

## 3. Mass Flow Uncertainty

The uncertainties contributing to the mass flow measurement have been quantified in Appendix A and now they will be combined. The uncertainty for flows between 20 L/min and 2000 L/min of nitrogen or argon in the 677 L tank is given in Table 1. The standard uncertainty of each sub-component is given in both relative (× 10^−6^) and dimensional forms. The units of the dimensional values are given in the third column. The relative contribution of each sub-component to the combined uncertainty is listed in the fourth column. This contribution is the percentage of the squared individual component relative to the sum of the squares of all sub-components. The uncertainty from the inventory volume, the combined uncertainty, the expanded uncertainty, and the uncertainty contributions are given as a range covering the minimum to maximum flow. To calculate their relative uncertainty in Table 1, the tank initial density was normalized by the tank final density and the inventory mass change was normalized by the total mass collected.

At the highest flow, uncertainty contributions are principally divided between the tank volume, the final gas density, and the inventory uncertainty. The uncertainty falls to 200 × 10^−6^ $\dot{m}$ for the smallest flows as the uncertainty contributions of the inventory volume become negligible. For an air flow measurement, the uncertainty of the 677 L system is less than 500 × 10^−6^ $\dot{m}$ over the entire flow range and the uncertainty is driven by the tank final density measurement (80 % contributor).

Table 2 presents the uncertainty of flow measurements from the 34 L system for flows between 1 L/min and 100 L/min. The expanded uncertainty varies between 270 × 10^−6^ $\dot{m}$ and 440 × 10^−6^ $\dot{m}$. At high flows, the significant uncertainty sources are the tank volume, the tank final density, and the uncorrelated inventory uncertainties. For low flows, the major contributors are tank volume and final gas density. For air flow measurements, the 34 L system has a nearly constant uncertainty over its entire flow range of about 500 × 10^−6^ $\dot{m}$ and it is driven by the uncertainty of the final gas density.

## 4. Experimental Validation of the Uncertainty Analysis

The performance of the flow standards and the validity of their uncertainty analyses (and particularly the assumed values for the uncorrelated inventory uncertainties) were checked by performing two types of experiments described in Secs. 4.1 and 4.2.

### 4.1 Comparison of the 34 L and 677 L Flow Standards

To test both systems, we performed comparisons between the two flow standards over the range of flows where they could both be used: 3 L/min < $\dot{m}$ < 110 L/min (3.6 g/min to 138 g/min of nitrogen). The collections ranged from as short as 18 s to more than 4 h.

**Fig. 10:**
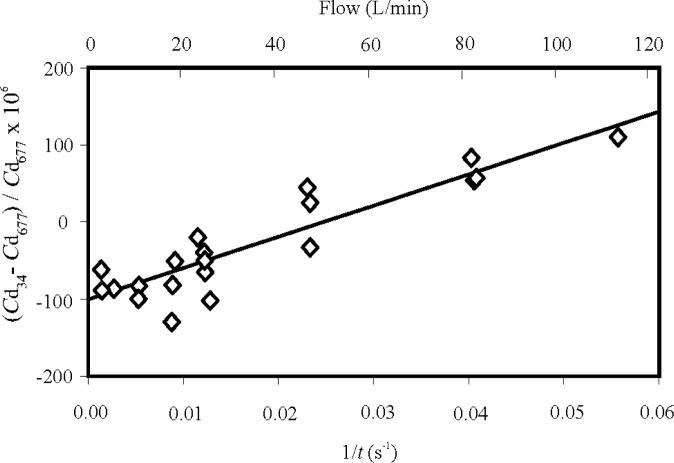
Difference in the discharge coefficient of critical flow venturis calibrated on both the 34 L and 677 L flow standards versus flow and the inverse of the collection time for the 34 L tank. Also plotted is a linear best fit of the data.

Figure 10 shows the difference in the discharge coefficients of several critical flow venturis as measured by both the 34 L and 677 L systems, plotted versus flow. The agreement between the two flow standards is 150 × 10^−6^ $\dot{m}$ over the entire range tested. The throat diameters of the venturis used for the comparisons ranged between 0.3 mm and 1.7 mm. The comparisons were done with the same pressure and temperature sensors associated with the venturi during the testing on both flow standards in order to reduce some possible sources of discharge coefficient differences. Numerous collections were made for each tank at each flow to confirm stability of the conditions at the meter under test.

How well should the two systems agree? The difference between the discharge coefficients measured by the two *PVTt* systems should be less than the root sum square (RSS) of the uncertainties of the two standards, especially when one considers that the uncertainties due to pressure and temperature measurements are correlated between the two standards. For the lowest flows of the comparison range, the uncertainties originating from the inventory volume are quite small for both systems and the observed differences between them are dominated by tank volume uncertainties. From Fig. 10 it can be seen that the two systems differ by about 100 × 10^−6^ $\dot{m}$ for flows less than 20 L/min. The RSS of the two relative standard volume uncertainties is 137 × 10^−6^ (*k* = 1).

At the higher flows of the comparison range, the uncertainties associated with the transient conditions in the inventory volume should be negligible in the 677 L system, but growing with increasing flow for the 34 L system. Because the collection times were 1/20th as long when using the smaller tank, any timing error (or, equivalently any imperfection of the mass cancellation technique) was 20 times more important when using the smaller tank. Figure 10 suggests that the inventory uncertainties cause the flow standard to read too high as the flow is increased, changing the difference by about 200 × 10^−6^ $\dot{m}$ over the range of flows compared. This value is comparable to the relative uncertainty of 135 × 10^−6^ (*k* = 1) contributed by the inventory volume at the highest flows in the 34 L system (61 % of 219 × 10^−6^ from Table 2). Therefore, the differences observed in the comparison are in reasonable accordance with the uncertainty analysis.

Figure 10 also shows the tank comparison results from the perspective of time measurement uncertainty rather than the mass. We interpreted the comparison results using the simplified model $\dot{m}=m/t$, where *m* is the mass collected and *t* is the collection time using the 34 L tank. Making the assumption that there exists a constant error in the mass measurement, *δm*, for all flows (say due to a tank volume error) and a constant error in time measurement, *δt*, for all flows (say due to the time constant of the inventory pressure sensor), we can derive a simple linear model for the error in mass flow measurement, i.e.,

$$\frac{\delta \dot{m}}{\dot{m}}=\frac{\delta m}{m}-\frac{\delta t}{t}.$$ (8)

Since the mass collected and the mass error are both essentially constant, this model suggests that a linear function of the inverse of the collection time should fit the tank comparison data. Such a linear function fits reasonably well, as shown in Fig. 10, and its slope implies a constant timing error of 4 ms. A timing error of this order is not surprising because the timing is based on a pressure sensor with a time constant of approximately 20 ms. The standard deviation of a measurement from the best fit line is 24 × 10^−6^ $\dot{m}$.

The comparison results could be the basis for corrections to the 34 L system. The intercept of Fig. 10 could be used to change the volume of the 34 L tank and improve the agreement between the two systems at low flows. The slope could be used to improve agreement at higher flows. This approach offers the possibility of reducing the comparison differences to zero with a standard deviation of 24 × 10^−6^ $\dot{m}$. However, these corrections have not been made at the present time for several reasons. The comparison results are consistent with the uncertainty analysis. Also, despite the success of Eq. (8), we feel that the inventory uncertainties are more related to pressure and temperature than time, so it is more appropriate to make improvements in those measurements to reduce the slope in the comparison data. The volume (or offset) differences can be improved by repeating and refining the volume expansion process used to determine the 34 L tank size. This approach adheres to the definition of primary standard for both systems since neither one has been calibrated by a flow measurement against some other flow standard.

During some of the comparison flows, we noticed that the pressure downstream of the critical flow venturi was significantly higher in the 34 L system than in the 677 L system (108 kPa vs 100 kPa) due to the smaller tube size and resultant higher pressure drop. For some of our venturis (with relatively short diffusers) this pressure difference caused slight changes in the upstream pressure (and the discharge coefficient), even at conditions well above the critical pressure ratio. Therefore, our assumption that for the same throat Reynolds number, the discharge coefficient of the venturi is independent of the downstream pressure may not be perfectly valid. We suspect that some of the differences observed between the tanks in Fig. 10 are due to the venturis (even though long diffusers were used).

In one series of experiments, the trigger pressure difference was purposely varied over a range from −2 kPa to 27 kPa at a constant flow of 82 L/min in the 34 L system. The purpose of the test was to measure the dependence of the venturi discharge coefficient on the trigger pressure difference and hence assess its influence on the inventory volume uncertainties. The tests showed a relative change of 10 × 10^−6^ in discharge coefficient for each 1 kPa change in the trigger pressure difference. Since the largest trigger pressure difference is less than 3 kPa in the present system, this effect is expected to contribute only 30 × 10^−6^ $\dot{m}$ to the flow uncertainty. Therefore the major contributor to the inventory uncertainty appears to be spatial inconsistency of the pressure and temperature fields between the start and stop diversions or some other, unknown flow dependent uncertainty source.

### 4.2 Multiple Diversions in the 677 L Flow Standard

To confirm that the uncertainty analysis for the inventory volume of the 677 L collection system was reasonable, we performed CFV calibrations at identical flows following two different protocols. In the first protocol, the inventory volume was dead-ended at the beginning and end of the collection interval, in the usual manner. In the second protocol, the collection interval was divided into two subintervals, i.e., each flow measurement had *two* start and stop diversions. The intermediate dead-end times were set up so that the pressure transients in the inventory volume still permitted the mass cancellation procedure. Breaking the collection into two subintervals has the effect of doubling the uncertainty contribution from the inventory volume. The CFV discharge coefficients from the two protocols were compared to assess the magnitude of the uncertainties introduced by the inventory volume and the flow diversion process. Three flows between 300 L/min and 1600 L/min were tested and the differences in discharge coefficient were all less than 75 × 10^−6^ $\dot{m}$ as shown in Table 3.

## 5. Conclusions

The design of a new gas flow standard composed of two *PVTt* collection tanks (34 L and 677 L) has been presented. The system is designed to calibrate critical flow venturis for flows from 1 L/min to 2000 L/min.

The flow standard has several novel features. The collection tanks are immersed in a water bath that matches the nominal room temperature and is stable and uniform to 1 mK. The collection tanks are divided into sections of small enough diameter that the gas inside them achieves thermal equilibrium with the surrounding water bath in 20 min or less. This reduces the contribution of temperature to the flow measurement uncertainty to a low level.

Uncertainties related to the inventory volume and the diversion of gas into the collection tank at the start and stop of a flow measurement have been studied in great detail. A thermodynamic model of the inventory volume during diversion has been utilized to understand the large pressure and temperature transients and the importance of sensor time constants on the flow measurement uncertainty. The flow standard is operated to achieve “mass cancellation” in the inventory volume, thereby taking advantage of correlated sensor uncertainties to minimize uncertainty contributions from the inventory volume. The uncertainty contributions of the inventory volume have been considered from both the time and mass perspectives.

The volumes of the collection tanks were measured by two methods, a gas gravimetric method and a volume expansion method. Six gravimetric determinations of the 677 L collection tank volume made with nitrogen and argon agree with a standard deviation of 16 × 10^−6^ *V*_T_.

A detailed uncertainty analysis for the gas flow standard has been presented in Appendix A. The analysis started with the basis equation utilized to calculate flow in a *PVTt* system and followed the propagation of uncertainties method suggested by international standards. The uncertainties of the sub-components have been examined at a fundamental level.

The uncertainty analysis shows that the 677 L system measures mass flow with an uncertainty between 200 × 10^−6^ $\dot{m}$ and 300 × 10^−6^ $\dot{m}$ for a pure gas like nitrogen or argon. The higher uncertainty applies to higher flows as the inventory transients and the related uncertainties grow larger. For the 34 L tank and pure gases, the uncertainties range from 440 × 10^−6^ $\dot{m}$ to 270 × 10^−6^ $\dot{m}$. The uncertainties are larger for the 34 L tank because the tank volume uncertainty is relatively greater and the inventory volume is relatively larger for the small system. For pure gas measurements, the largest sources of uncertainty can be traced to pressure measurement (about 70 × 10^−6^ *P*) which is the major contributor to gas density and tank volume uncertainties. For air flow measurements using gas from the existing compressor and drier, mass flow uncertainties are about 500 × 10^−6^ $\dot{m}$ for both standards and the major contribution is the uncertainty in the moisture content of the air.

Comparisons between the 34 L and 677 L standards from 3 L/min to 100 L/min show agreement within 150 × 10^−6^ $\dot{m}$ or better. Experiments using single diversions (normal operation) and double diversions to the collection tank were used to validate the uncertainty estimates of the 677 L inventory volume and the differences between these two methods were less than 75 × 10^−6^ $\dot{m}$. The evaluation results along with comparisons to previously existing gas flow standards support the uncertainty statements for the new standards.

There are opportunities for improvement in the uncertainty of the new *PVTt* flow standards. Pressure uncertainties are the most significant contributors, through the tank volume determinations as well as through the final gas density measurement. Therefore, tank pressure sensors with better calibration stability over time will be sought. Faster inventory pressure sensors would improve the mass cancellation procedure and reduce the inventory uncertainties.

## Figures and Tables

**Table 1:** Uncertainty of nitrogen flow measurement with the 677 L standard

Uncertainty category	Standard uncertainty (*k* = 1)		Contrib	Comments
Flow (677 L, N_2_)	Relative (×10^6^)	Absolute	(%)	
Tank volume	71	48.44 cm^3^	50 to 23	see Table A12
Tank initial density	10	2.27 × 10^−12^ g/cm^3^	1 to 0	
Tank final density	68	7.82 × 10^−8^ g/cm^3^	45 to 21	see Table A6
Inventory mass change	0 to 109	0.084 g	0 to 53	see Table A18
Collection time	5	0.3 ms	0 to 1	see Table A8
Std deviation of repeated meas.	20	0.001 g/s	4 to 2	
RSS (combined uncertainty)	102 to 150			
Expanded uncertainty (*k* = 2)	204 to 300			

**Table 2:** Uncertainty of nitrogen flow measurement with the 34 L standard

Uncertainty category	Standard uncertainty (*k* = 1)		Contrib	Comments
Flow (34 L, N_2_)	Relative (×10^6^)	Absolute	(%)	
Tank volume	116	3.95 cm^3^	72 to 28	see Table A13
Tank initial density	10	2.27 × 10^−12^ g/cm^3^	1 to 0	
Tank final density	68	7.82 × 10^−8^ g/cm^3^	25 to 10	see Table A6
Inventory mass change	0 to 170	0.007 g	0 to 61	see Table A19
Collection time	15	0.3 ms	0 to 0	see Table A8
Std deviation of repeated meas.	20	4 × 10^−5^ g/s	2 to 1	
RSS (combined uncertainty)	137 to 219			
Expanded uncertainty (*k* = 2)	274 to 438			

**Table 3:** Differences in CFV discharge coefficients (*C*_d_) for two and one diversion in the 677 L flow standard

Flow (L/min)	[*C*_d_ (2 diversions) – *C*_d_]/*C*_d_ × 10^6^
300	53 ± 25
700	−27 ± 31
1600	75 ± 122

**Fig. A1:**
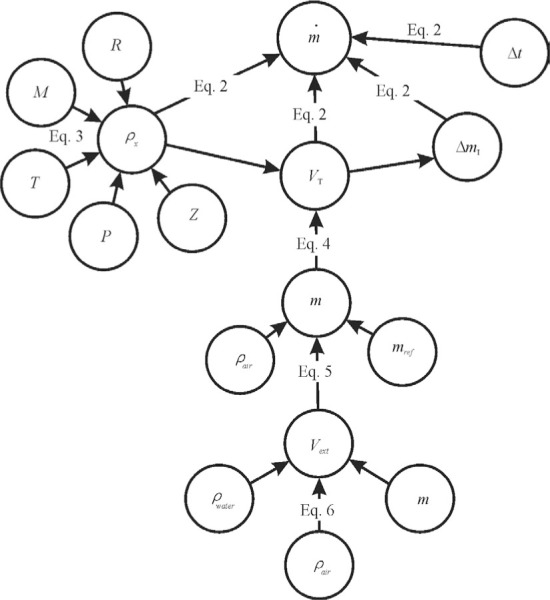
The chain of measurements and equations used for the *PVTt* flow standard.

**Fig. A2:**
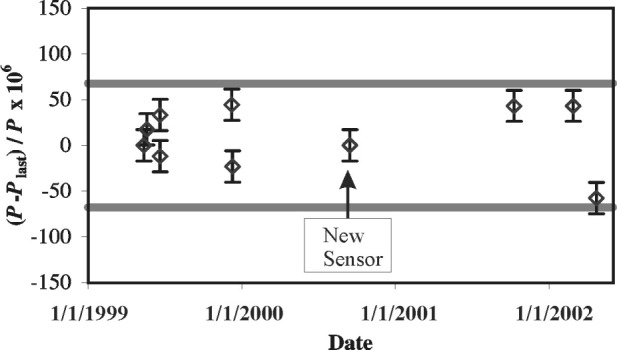
A calibration control chart for a 200 kPa pressure transducer used to measure the collection tank pressure.

## 6. Appendix A. Uncertainty Analysis

The uncertainty of a mass flow measurement made with the *PVTt* standard will now be considered following the propagation of uncertainties techniques described in the ISO Guide to the Expression of Uncertainty in Measurement [5]. The process identifies the equations involved in the flow measurement so that the sensitivity of the final result to uncertainties in the input quantities can be evaluated. The uncertainty of each of the input quantities is determined, weighted by its sensitivity, and combined with the other uncertainty components to arrive at a combined uncertainty.

As described in the references [5,11], consider a process that has an output, *y*, based on *N* input quantities, *x_i_*. For the generic basis equation:

$$y=y(x_{1},x_{2},\ldots ,x_{N}),$$ (A1)

if all the uncertainty components are uncorrelated, the standard uncertainties are combined by root-sum-square (RSS):

$$u_{c}(y)\sqrt{\sum_{i=1}^{N}\left(\frac{\partial y}{\partial x_{i}}\right)u^{2}(x_{i})},$$ (A2)

where *u*(*x_i_*) is the standard uncertainty for each of the inputs, and *u*_c_(*y*) is the combined standard uncertainty of the measurand. The partial derivatives in Eq. (A2) represent the sensitivity of the measurand to the uncertainty of each input quantity

In cases where correlated uncertainties are significant (as in the following analysis), the following expression should be used instead of Eq. (A2):

$$u_{c}(y)=\sqrt{\sum_{i=1}^{N}{\left(\frac{\partial y}{\partial x_{i}}\right)}^{2}u^{2}(x_{i})+2\sum_{i=1}^{N-1}\sum_{j\neq +1}^{N}\frac{\partial y}{\partial x_{i}}\frac{\partial y}{\partial x_{j}}u(x_{i})u(x_{j})r(x_{i},x_{j})},$$ (A3)

where *r*(*x_i_*, *x_j_*) is the correlation coefficient, ranging from −1 to 1, and equaling zero if the two components are uncorrelated. As will be seen in the following analysis, some uncertainty components in the present system are correlated and this leads to a significant improvement in the uncertainty of the measurand.

A simple example of a correlated uncertainty is illustrative. Suppose that a thermometer was used to measure a temperature difference. Also suppose that the only uncertainty in the thermometer measurement was an unknown offset in its calibration. When the difference between two temperatures was calculated from measurements made with this thermometer, the offset would cancel and would not contribute to the uncertainty of the temperature difference. In this case, the subtraction process used to calculate the difference leads to sensitivity coefficients of opposite sign for the two temperature measurements. Since the sensor always has the same offset, the uncertainties are perfectly correlated [*r*(*x_i_*, *x_j_*) = 1]. When this hypothetical scenario is processed through Eq. (A3), the uncertainty of the temperature difference is zero. Of course in a real case, there would be other, uncorrelated uncertainties that would make the uncertainty of the temperature difference non-zero. Nonetheless, the example demonstrates that under certain circumstances, correlated uncertainties will reduce the uncertainty of a measured quantity.

In the following uncertainty analysis, correlated and uncorrelated uncertainties will be treated as separate components, even if they are related to the same physical quantity. For instance, there will be a correlated as well as an uncorrelated inventory pressure component. In this manner, the correlated components can be considered as having a correlation coefficient of 1, while uncorrelated components have correlation coefficients of 0. This approach simplifies the process to deciding which uncertainty sources are correlated versus uncorrelated and checking that the assumption of perfect correlation is reasonable.

Most of the equations utilized to calculate flow, mass, volume, and other necessary intermediate quantities for the *PVTt* standard have been discussed in prior sections. In Fig. A1 the information is summarized in a diagram that shows the measurement chain used to calculate flow. At the top of the diagram is the output, mass flow. At the second level are the inputs to Eq. (2), the quantities needed to calculate mass flow: density, volumes, collection time, etc. To calculate density, the inputs to Eq. (3) are necessary: pressure, temperature, compressibility, the universal gas constant, and molecular weight. The other necessary quantities and their basis equations are shown as well. Figure A1 will serve as a guide for the *PVTt* uncertainty analysis.

The discharge coefficient resulting from a flowmeter calibration will have additional uncertainties not considered herein due to measurements associated with the meter under test. For instance, if the meter under test is a critical flow venturi, uncertainties related to the temperature and pressure measurements at the meter must be included in the uncertainty of the discharge coefficient.

The uncertainties tabulated herein are *k* = 1, standard, or 68 % confidence level uncertainties. At the conclusion of the uncertainty analysis, a coverage factor of 2 will be applied to give an expanded uncertainty for mass flow measurements with an approximate 95 % confidence level. In the remainder of Sec. 6, the uncertainty components that contribute to the *PVTt* mass flow measurement will be traced to their most fundamental sources.

### 6.1 Pressure

A Ruska Model 2465–754^1^ gas lubricated piston pressure gauge is used as the primary pressure standard to calibrate pressure transducers within the Fluid Flow Group. The uncertainties in a single pressure measurement made with this device are listed in Table A1. Uncertainties in the pressure standard can be traced to the effective area of the piston, piston thermal expansion, the masses, local gravity, and the measurement of the pressure under the bell jar covering the piston and masses (necessary for absolute pressure measurements). The uncertainty shown in Table A1 is for a pressure value of 100 kPa. No buoyancy corrections are made to the masses since the reference pressure, and hence the density under the bell, are small enough that the buoyancy corrections (and uncertainties) are negligible (≪1 × 10^−6^ *P*). The uncertainty of the piston pressure gauge is 17 × 10^−6^ *P* at 100 kPa.

#### 6.1.1 Collection Tank Pressure

Measurements of the collection tank initial pressure are made with a pair of thermocouple vacuum gauges (Varian Convectorr P-type) that have been calibrated by comparison to a reference standard in the NIST Pressure and Vacuum Group. The manufacturer’s uncertainty specification for this gauge is 10 % of reading. Based on the NIST calibration results, the consistent agreement between the redundant sensors, and the repeatable readings of the gauges at the vacuum pump ultimate pressure, a standard uncertainty of 5 % of reading will be used. As will be seen when the components are combined to give the flow measurement uncertainty, this large value has little impact due to the low initial pressure in the collection tank (20 Pa).

Pressure measurements of the full collection tank are made with a Paroscientific Model 740 with a full scale of 200 kPa. The manufacturer’s uncertainty specification for this transducer is 0.01 % of full scale, but under the conditions of the present usage, the uncertainty is less. The uncertainties in the collection tank pressure measurement are listed in Table A2. They include the uncertainties from the piston pressure gage, the long term drift of the Paroscientific transducer which has been quantified by periodic re-calibrations, as well as the residuals from the best fit calibration equation (including hysteresis), and thermal effects. Uncertainties due to spatial non-uniformity of pressure within the tank and time response of the sensor are negligible since the calibration procedure is to wait as much as 20 min for equilibration before the measurements are made.

Figure A2 is a control chart that shows the changes in pressure calibration (×10^−6^) vs time at a pressure of 100 kPa for one of the pressure transducers used to measure collection tank pressure. Also shown are the *k* = 1 uncertainty tolerance bounds (64 × 10^−6^ *P* from Table A2) and error bars that represent the *k* = 1 uncertainty of the piston pressure gauge used to calibrate the sensor (17 × 10^−6^ *P* from Table A1).

Temperature effects as large as 40 × 10^−6^ *P* have been observed in the tank pressure sensor and care was taken to minimize their influence. When the tank is quickly filled from a pressurized cylinder during the volume determination process, cold gas enters the sensor, cooling it. The pressure readings asymptotically approach a final value as the sensor returns to room temperature (with a time constant of approximately 1 hr). The temperature dependence of the sensor was confirmed by testing with an environmental chamber. Temperature effects also result in a hysteresis loop for the sensor calibration data that enlarges the calibration fit residuals. The calibration process entails increasing and decreasing the pressure in steps. The pressure steps result in heating and cooling of the pressure sensing elements and a hysteresis loop. Therefore the residuals of the pressure calibration fit include contributions due to thermal effects. We noticed that the thermal effects due to pressure changes in the transducers can be larger than the values given in Table A2. During volume determinations, we allowed sufficient time for the sensor to return to room temperature so that the remaining temperature effects were much smaller than the allowance for calibration drift.

### 6.2 Temperature

The temperature sensors used in the flow standard are traceable to the NIST Thermometry Group through calibrations made with a four-wire thermister transfer standard (Thermometrics Model TS8901) and a recirculating water constant temperature bath. The uncertainty of the transfer standard thermister is 1.2 mK (see Table A3). The drift of the transfer standard is considered negligible based on seven annual calibrations that have always differed from each other by less than the calibration uncertainty.

#### 6.2.1 Collection Tank Temperature

The measurement of the temperature of the gas in the collection tank has additional uncertainties that are listed and quantified in Table A4. Temperature is measured with YSI Model 46000 thermisters in 3 mm diameter stainless steel sheaths, a Keithley model 224 current source, a Keithley model 7001 switch system, and a Keithley model 2002 multimeter. Uncertainty sources include the temperature transfer standard covered by Table A3, the uniformity and stability of the water bath used to calibrate the thermisters, and the residuals of the best-fit equation to the calibration data. The largest uncertainty component is the calibration drift between periodic calibrations. Radiation and stem conduction are negligible since the thermisters are immersed at least 15 cm in room temperature water. Tests were conducted to measure the significance of self-heating by varying the thermister current while the sensor was held in a stable water bath and watching the resulting change in sensor reading. Based on this experiment, the current through the 5000 Ω thermisters was set to 10 µA which leads to self-heating of less than 1 mK. The *PVTt* bath stability and uniformity (1 mK) were discussed earlier, as was the issue of thermal equilibrium between the water bath and the gas in the collection tank. The sensors are calibrated over their entire range of usage, so there is no uncertainty related to extrapolation of their calibration data. Uncertainty related to the time response of the thermisters is negligible since the time constant for the sensor is on the order of 10 s and the wait for thermal equilibrium is 30 or more times longer.

Small portions of the gas collection tank are not immersed in the water bath. They include the tubing connecting the outlet of the diverter valve to the collection tank, the tubing that connects the tank to pressure and vacuum transducers, and the internal volume of these transducers. Because we assumed that the bath temperature represents the gas temperature, and the room temperature may differ from that of the bath, the small portion of the tank not immersed leads to a gas temperature uncertainty. The room temperature is maintained at 23.5 °C ± 1 °C. The fractional error in mass contained in the collection tank due to a 1 K difference between the room and the water bath is:

$$\frac{\delta m}{m}\approx \frac{V_{\text{out}}}{V_{T}}\frac{\delta T}{T},$$ (A4)

where *V*_out_ is the volume of the portion of the tank that is at room temperature and *δT* is the difference between the room and water bath temperatures. For the large tank, *V*_out_ is 200 cm^3^ and the total tank volume is 677 L. For *δT* of 1 K, the relative mass uncertainty is 1 × 10^−6^. This uncertainty will be treated as an uncertainty in the average gas temperature of 1 × 10^−6^ *T* or 0.3 mK in the following analysis. For the small tank this temperature uncertainty is 1.2 mK or 4 × 10^−6^ *T*.

By RSS of the components listed in Table A4, the combined uncertainty for the average temperature of the gas in the collection tank is 7 mK when using the thermisters dedicated to the flow standard.

### 6.3 Compressibility, Molecular Weight, and Gas Constant

The remaining contributors to gas density uncertainty are the compressibility, the molecular weight, and the gas constant. Four gas cases will be considered. Tank volume determinations were conducted with ultra high purity nitrogen (99.999 % N_2_) and ultra high purity argon (99.999 % Ar). During normal calibrations, industrial liquid nitrogen vaporized from dewars (99.998 % N_2_) and compressed, dried air are used.

#### 6.3.1 Gas Constant

The universal gas constant is known with relative standard uncertainty better than 2 × 10^−6^ [12].

#### 6.3.2 Compressibility

The compressibility factor can be calculated from the following expression:

$$Z=1+B\rho_{n}+C{\rho_{2}}^{2},$$ (A5)

where *ρ_n_* is the molar density (mol/cm^3^) and *B* and *C* are the second and third virial coefficients respectively. For nitrogen, the virial coefficients can be calculated from the correlations:

$$B_{N_{2}}=-131.21+0.65125T-7.636\times 10^{-4}T^{2},$$ (A6)

$$C_{N_{2}}=3454.2-11.35T+0.015T^{2},$$ (A7)

where temperature is in K and *B* and *C* are in cm^3^/mol and cm^6^/mol^2^, respectively, and the units of the constants have been surpressed. For dry air the correlations for the virial coefficients are:

$$B_{\text{Air}}=-137.06+0.66785T-7.833\times 10^{-4}T^{2},$$ (A8)

$$C_{\text{Air}}=3528.9-12.40T+0.016T^{2},$$ (A9)

These expressions are the result of least squares best fitting to outputs from a database of the property measurements by numerous experimenters [13]. Equations (A6) through (A9) were fitted to data over the range of 270 K to 330 K. This range allows application of the correlations at the conditions found in the test section of the flow standard. However, it should be noted that the conditions in the collection tank are much narrower, with pressures ranging from 0 kPa to 101 kPa at a nearly constant temperature of 296.5 K. For these narrower conditions, the third virial coefficient could be ignored with negligible impact on the density uncertainty.

Uncertainty estimates for experimental studies of compressibility are often unavailable, especially for older publications. Comparison of previously compiled compressibility values obtained by various researchers [14,15,16,17] shows agreement to within 10 × 10^−6^ in the 270 K to 330 K temperature range at 100 kPa. Perhaps more valuable is that this level of agreement is achieved between compressibility measurements made by the traditional *pVT* method and by the more recent speed of sound techniques [18]. Based on this information, a relative standard uncertainty of 10 × 10^−6^ will be used for the experimental measurements of compressibility. This uncertainty is for a pressure of 100 kPa and it scales with density. The residuals of the equation fitting process to the experimental data are negligibly small (<1 × 10^−6^ *Z*). The uncertainty components of the compressibility factor are listed in Table A5.

The source of the dry air used in the flow standard is a Joy oil-free two stage reciprocating compressor and a Zurn refrigeration dryer. The mole fraction of water in this air source is 0.0010 ± 0.0005. This uncertainty in composition leads to uncertainty in the compressibility of only 1 × 10^−6^ *Z*. The uncertainty in compressibility due to impurities is smaller for the other gases.

#### 6.3.3 Molecular Weight

The departure of the molecular weight of ultra high purity nitrogen, industrial liquid nitrogen, and ultra high purity argon from the molecular weight of the pure substance was examined using the impurity specifications of the gas manufacturer. This analysis results in molecular weight relative standard uncertainties less than 1 × 10^−6^, but 1 × 10^−6^ will be assumed for the molecular weight of nitrogen (28.01348 g/mol) and argon (39.94800 g/mol). For the dry air described above, the molecular weight is 28.9537 g/mol and its relative standard uncertainty is 190 × 10^−6^ due to the variability of water content.

### 6.4 Density

Now that the sub-components have been quantified, the uncertainty of density measurements made in the collection tank with nitrogen and argon (for volume determinations and for flow measurements) and with dry air (for flow measurements) can be calculated and they are presented in Tables A6 and A7. For the pure gases, the relative standard uncertainty is 68 × 10^−6^ and the primary contributor is the pressure measurement. For air, the relative standard uncertainty is 208 × 10^−6^ and the largest contribution is due to water content variations. The uncertainties of the density of ambient air (needed for buoyancy corrections) and of distilled water are considered in the section pertaining to tank volume determinations.

### 6.5 Collection Time

As explained in prior sections, the collection time is an approximate time measured by timers that is then corrected via analysis of records of the inventory pressure data and trigger voltages to minimize inventory mass and improve uncertainty cancellation. The base time is measured redundantly with two Hewlett-Packard Model 53131A counters. The counter calibration and usage leads to less than 0.01 ms uncertainty. Due to the 3000 Hz recording frequency of the inventory pressure and trigger data, the actual rise of the trigger voltage can be any time within a 0.33 ms window. Assuming a rectangular distribution, the post-processing corrections will have a standard uncertainty of 0.19 ms. This uncertainty applies to both the start and stop times. The time uncertainties of the two pressure measurements used in the mass cancellation procedure are negligible since the times are found by interpolation of the data records and are much smaller than 0.33 ms. The combined collection time uncertainty is 0.3 ms (see Table A8).

### 6.6 Volume of the 677 L Collection Tank (Gravimetric Method)

The uncertainty of the determination of the collection tank volume by the previously described gravimetric method is traceable to the uncertainty of the mass and density measurements made during the process, which are in turn dependent on the quantities shown in Fig. A1. The uncertainty of the density measurements for the pure nitrogen and argon gases used for the volume measurements was given in Table A6. The scale used was a Mettler-Toledo model PR10003, which has a 10 kg capacity. The uncertainty of the mass measurements of the high pressure cylinder before and after discharge is dependent on the buoyancy corrections (ambient air density and cylinder external volume), the reference masses used with the mass comparator, and the performance of the mass comparator. The measurement of the external volume of the high pressure cylinder via the Archimedes principle was described in an earlier section. The uncertainty of this measurement is traceable to the density of distilled water, ambient air, reference masses, and the performance of the mass measuring systems.

The uncertainty of the density of ambient air during the course of the various weighing procedures is given in Table A9. The pressure, temperature, and relative humidity uncertainties account for instrument calibration uncertainties as well as variations in the room conditions during the time needed to make a mass measurement. The relative combined uncertainty of the ambient air density is about 500 × 10^−6^, with the largest contribution being from temperature.

The uncertainties related to the determination of the external volume of the high pressure cylinder are listed in Table A10. The 1 × 10^−6^ relative standard uncertainty for the apparent mass in air is based on uncertainty calculations for the true mass of the weighed cylinder (discussed later). The uncertainty of the apparent mass in water is based on the recorded temperatures of water which made the cylinder barely sink or barely float. Uncertainty of the water temperature for neutral buoyancy of 0.2 K leads to a value for the uncertainty of the apparent mass in water. This uncertainty is not reported in a relative form since the apparent mass in water is zero and the relative uncertainty is therefore undefined. The RSS is calculated using the results in the third column and their sensitivity coefficient. The water density uncertainty includes thermometer uncertainties, estimates of non-uniformity of the water temperature, and uncertainty of the water density correlation obtained from the literature. As previously stated, different values of external volume were used for the full and empty cylinder due to pressure dilation, but an uncertainty related to pressure and temperature effects is included here. The collection tank volume is not very sensitive to this external tank volume, hence larger uncertainty values than 51 × 10^−6^ *V*_ext_ could certainly be tolerated.

The relative standard uncertainties of the cylinder mass measurement (Table A11) are quite small (1 × 10^−6^) with the major components being the reference masses, the room air density (for buoyancy corrections) and the performance of the comparator (repeatability of the five measurements made with the comparator, a type A uncertainty). The complete mass measurement process was repeated several times for the full and empty cylinder conditions to assess the repeatability of the process, and these repeated mass values never differed by more than 1 × 10^−6^ *m*_c_. This repetition was undertaken since a previously used cylinder showed changes over time, probably due to absorption of water from room humidity variations. The uncertainty of the density of the reference masses (7.8 g/cm^3^) is negligible since the sensitivity of mass measurements to this component is extremely small. While some of the mass measurement uncertainties for the full and empty cylinder are correlated and this could be used to reduce the uncertainty of the mass delivered to the collection tank, this benefit was not utilized since the improvement was not significant. A table of uncertainty components of the empty cylinder mass measurement is not presented because it is so similar to Table A11.

Finally, the uncertainty of the collection tank volume is presented in Table A12. The relative value of the initial collection tank density uncertainty is quite large, but the sensitivity of the result to this quantity is very small. Recall that uncertainty due to the effects of room temperature variations on the portion of the collection tank not submerged has already been incorporated as a temperature uncertainty. The effects of pressure changes from vacuum to 100 kPa on the tank volume have been considered analytically and found to be negligible. A small volume (1 cm^3^) of connecting tubing and valve was necessary to introduce gas from the pressurized cylinder to the collection tank. This volume was measured with alcohol and a graduated syringe and the uncertainty of this volume correction was estimated to be 0.5 cm^3^. By far the largest contribution is from the final gas density, and this in turn is nearly completely traceable to the pressure measurement.

Each gravimetric volume determination required one day to complete due to the time required to achieve ultimate vacuum in the tank (1 Pa), the time for the pressure transducer to reach thermal equilibrium after filling, and the time to take multiple cylinder mass measurements separated by an hour each time. The volume measurements were done six times, four times with nitrogen and twice with argon. The standard deviation of these six determinations was 16 × 10^−6^ *V*_T_, quite good when one considers that two different gases were used and the uncertainty of their equations of state. The combined uncertainty of the 677 L collection tank volume is 71 × 10^−6^ *V*_T_. The 677 L volume was used as a reference to determine the remaining three unknown volumes via the volume expansion method.

### 6.7 Volume of the 34 L Collection Tank (Volume Expansion Method)

As explained in a prior section, the volume expansion method is performed by pressurizing a known volume with pure gas and opening a valve to expand the gas into a previously evacuated unknown volume. The density changes and known volume are used to calculate the unknown volume via Eq. (7). In this case the 677 L volume was used to determine the large inventory volume and the 34 L collection tank volume. Subsequently, the 34 L collection tank volume was used to determine the small inventory volume.

Significant uncertainties of the volume expansion method are related to density and the measurement of pressure. Many components of the previously given pressure uncertainty are correlated for a pressure change measurement made with the same pressure transducer over a short time period (about 1 h). Uncertainties of the piston pressure gauge and the sensor drift can be considered correlated for the short time period and small pressure changes involved (5 kPa to 8 kPa out of 200 kPa full scale).

The uncorrelated uncertainties in the measurement of pressure change are due to sensor non-linearity, resolution, hysteresis, and thermal effects. In order to quantify these uncertainties for the volume expansion method, the piston pressure gauge was used to repeatedly provide a reference step change of 6 kPa to the pressure transducer. The pressure change measured by the pressure transducer was compared to the pressure change calculated from the pressure standard. Based on these experiments, the uncertainty of the pressure difference measurement during the 34 L tank volume determination is 0.3 Pa

The 34 L tank volume uncertainty is summarized in Table A13. The largest uncertainty contributions are from the 677 L volume uncertainty, the density change in the 34 L volume, and the standard deviation of the repeated volume measurements. The uncertainty of the density change in the 677 L volume is dominated by uncertainty in the measurement of the pressure change.

### 6.8 Inventory Volume

The mass change in the inventory volume is negligible since we used the mass cancellation procedure. However, since there are imperfections in the procedure, uncertainty components related to the inventory mass change cannot be neglected. Fortunately, the most significant of these uncertainty components (due to sensor time constants) are correlated between the start and stop diversions for the methods of operation used in this flow standard. Other correlated inventory volume uncertainties include the pressure and temperature sensor calibrations and the differences between sensed and stagnation values of pressure and temperature. For instance, the inventory temperature is measured incorrectly low by the same amount at both the start and stop conditions due to slow sensor response time, so cancellation of temperature uncertainty occurs. More precisely, the uncertainty of the mass change within the inventory volume caused by the *correlated* pressure and temperature uncertainties can be expressed as:

$$u(\Delta m_{I})=\frac{V_{I}M}{ZR}{\left[{\left(\frac{1}{T_{I}^{f}}u(P_{I}^{f})-\frac{1}{T_{I}^{i}}u(P_{I}^{i})\right)}^{2}+{\left(\frac{P_{I}^{i}}{{\left(T_{I}^{f}\right)}^{2}}u(T_{I}^{f})-\frac{P_{I}^{i}}{{\left(T_{I}^{i}\right)}^{2}}u(T_{I}^{I})\right)}^{2}\right]}^{1/2}$$ (A10)

where in this equation, *u*(*P*_I_), *u*(*T*_I_), and *u*(Δ*m*_I_) are the uncertainties of the inventory pressure, the inventory temperature, and the inventory mass change during the collection, respectively. Note that if the uncertainties and the initial and final conditions are equal (i.e., $u(T_{I}^{i})=u(T_{I}^{f})$, $u(P_{I}^{i})=u(P_{I}^{f})$, $T_{I}^{i}=T_{I}^{f}$ and $P_{I}^{i}=P_{I}^{f}$), then the terms within parentheses cancel, and the flow uncertainty related to the inventory volume is zero. Equation (A10) demonstrates the benefit of matching the initial and final inventory conditions to optimize the cancellation of correlated uncertainties.

Not all of the measurement uncertainties of the inventory volume are correlated. For perfect inventory mass cancellation, the pressure and temperature measured at specific locations in the inventory volume must exhibit perfect correlation with the pressures and temperatures throughout the inventory volume. In this way, the sensor readings “represent” the conditions throughout the volume and when the readings at the specific locations match, the conditions throughout the inventory volume are matched. Unfortunately, this representative relationship may not exist and there may be inconsistencies between the pressure and temperature fields between the start and stop diversions. These inconsistencies may originate from a change in the inventory wall temperature or from differences in the flow paths between the start and stop diversions. The spatial inconsistencies are uncorrelated and their magnitude is likely a function of the mass flow.

Another source of inventory uncertainty, alluded to previously, is due to imperfection in matching the stop diversion pressure to the start diversion pressure. Recall that the inventory pressure is recorded while the bypass valve is open (nominally the barometric pressure), and that the tank filling is stopped when the pressure reaches this same pressure. In this way, the pressure at the beginning of the dead-end time transients is nearly equal for both diversions and the symmetry of the transients is improved. At high flows, it becomes more difficult to match these initial pressures in the high speed data records and a “trigger pressure difference” that increases with increasing flow occurs. The size of the trigger pressure difference can be reduced by using faster sensors and a fast data acquisition and diverter valve control system. An example of the trigger pressure difference can be seen in Fig. 7, where at times less than zero, the pressure traces differ by about 1.6 kPa. The trigger pressure difference, coupled with the “historical” nature of the inventory pressure measurements due to the sensor time constant, is another reason that matching of the pressure sensor readings does not necessarily lead to matching of the actual conditions in the inventory volume. Hence the trigger pressure difference is another source of inventory uncertainty that scales with the flow.

It is difficult to assess the magnitude of the uncorrelated uncertainties of pressure and temperature between the start and stop diversions in the inventory volume. Our strategy is to estimate the uncorrelated inventory uncertainties and then perform experiments (see Sec. 4) to confirm that the estimates are reasonable. We assumed that the uncorrelated inventory uncertainties scale with the flow and are essentially zero at the minimum flow of each tank. We will assume values of 3 kPa and 9 K (about 3 % of the nominal values) for the maximum flow of each tank.

Uncertainties related to the fast measurement of pressure with a 700 kPa full scale Heise Model HPO sensor in the inventory volume are listed in Table A14, separated into correlated and uncorrelated components.

The uncertainties of the measurement of temperature in the inventory volume are listed in Table A15, again divided into correlated and uncorrelated components. The uncertainties include calibration, time response, sensed versus stagnation issues, sensor repeatability, and spatial non-uniformity or inconsistency between start and stop diversions.

The uncertainties for the large and small inventory volumes determined by the volume expansion method are presented in Tables A16 and A17. Although the relative uncertainties of these volumes are large, the sensitivity of the flow measurement to these volumes is small due to the inventory mass cancellation scheme and the relatively small size of *V*_I_. For the very small pressure changes used for the inventory volume determinations, the ability of the pressure transducers to measure the pressure change becomes a large uncertainty issue. The standard deviation of repeated measurements is large for these volume determinations due to the uncertainty of the small pressure change measurements. The ratio of the known tank volume to the unknown inventory volume is about 500 to 1, hence the pressure change is only about 0.3 kPa for an initial pressure of 150 kPa. The uncertainty of the pressure change measurement (0.3 Pa) is based on the previously described experiments using the pressure standard. The instrument resolution (1.3 Pa) is also a concern for such small pressure changes measured with a 200 kPa full scale transducer. Although the noise in the pressure data is greater than 1.3 Pa, averaging the pressure measurements over 30 s reduces the resolution to an acceptable level. For the inventory volumes, the “extra volume” includes tubing and valves used to introduce gas to the system and volume changes caused by the actuation of the diverter valves. The extra volume corrections are based on liquid transfer into or out of the volume by pipette, and/or by dimensional calculations.

Tables A18 and A19 show the uncertainty in the mass change in the inventory volume for the largest flows in the 34 L and 677 L systems. The relative uncertainty of Δ*m*_I_ is undefined since the mass change is zero.

The uncertainties of gas density, volume, collection time, and inventory mass change provided in this Appendix have been combined in Sec. 3 to give the uncertainty in mass flow.

**Table A1:** Uncertainties for a 100 kPa pressure measurement made with the piston pressure gage used as the standard for pressure calibrations

Uncertainty category	Standard uncertainty (*k* = 1)		Contrib	Comments
Piston Pressure gage	Relative (×10^6^)	Absolute (kPa)	(%)	
*Pressure value*		100		
Piston effective area	12	0.0012	65	From NIST Pressure and Vacuum Group cal.
Thermal expansion	6	0.0006	4	Assume *T* unc of 0.2 K
Masses	1	0.0001	0	Mass Group calibration
Local gravity	0.2	0.00002	0	9.801011+/−0.000002
Air density for buoyancy	0	0	0	Neg. air density relative to mass density
Ref. *P* for absolute	10	0.001	31	Based on calibration data of the vac gauge
RSS	17	0.0017		

**Table A2:** Uncertainties in the collection tank pressure measurement at 100 kPa

Uncertainty category	Standard uncertainty (*k* = 1)		Contrib	Comments
Pressure measurement	Relative (×10^6^)	Absolute (kPa)	(%)	
*Pressure value*		100		
Piston pressure gage	17	0.0017	7	From Table A1
Drift	60	0.0060	88	<0.01 % in 6 months, assume rect.
Residuals, hysteresis, thermal effects	14	0.0014	5	From cal. records, experiments
RSS	64	0.0064		

**Table A3:** Uncertainties for the Fluid Flow Group temperature transfer standard

Uncertainty category	Standard uncertainty (*k* = 1)		Contrib	Comments
Temperature transfer standard	Relative (×10^6^)	Absolute (mK)	%	
*Temperature value*		3 × 10^5^		
Thermometry Group cal	4	1.2	94	Unc. for 274 K to 368 K
Fit residuals	1	0.3	6	Some years, 1/6 this size
Drift	0	0	0	Less than discernable given cal unc.
Radiation, self-heating, etc.	0	0	0	Deeply immersed in water bath
RSS	4	1.2		

**Table A4:** Uncertainty of average gas temperature in the collection tank with the dedicated temperature sensors

Uncertainty category	Standard uncertainty (*k* = 1)		Contrib	Comments
Tank gas average temperature	Relative (×10^6^)	Absolute (mK)	(%)	
*Temperature value*		297000		
Temperature transfer standard	4	1.2	4	From Table A3
Cal. bath uniformity and stability	3	1	2	Based on notes made during cal
Fit residuals	7	2	9	
Drift (I, R, DMM, thermistors)	20	5.8	77	YSI spec is <10mK/10 months, assume rect.
Radiation, stem cond., self-heating	3	1	2	Expt. varied current
*PVTt* bath uniformity and stability	3	1	2	See Fig. 4
Diff. between water and room T’s	4	1.2	3	See Eq. (A4)
Extrapolation	0	0	0	Cal covers whole operating range
Sensor time constant	0	0	0	Wait time is >10 × sensor time constant
RSS	22	7		

**Table A5:** Uncertainty of the compressibility for nitrogen, argon, and dry air

Uncertainty category	Standard uncertainty (*k* = 1)		Contrib	Comments
Compressibility (*Z*)	Relative (×10^6^)	Absolute	(%)	
*Compressibility value*		1		
Experimental data	10	1 × 10^−5^	99	Based on analysis of literature
Impurity effects on *Z*	1	1 × 10^−6^	1	
Residuals of best fit	0	0	0	<1 × 10^−6^
RSS	10	1 × 10^−5^		

**Table A6:** The uncertainty of collection tank gas density for nitrogen and argon

Uncertainty category	Standard uncertainty(*k* = 1)		Contrib	Comments
Collection tank density (N_2_ & Ar)	Relative (×10^6^)	Absolute	(%)	
Pressure	64	6.45 × 10^−3^ kPa	88	From Table A2
Temperature	22	6.47 mK	10	From Table A4
Compressibility	10	1 × 10^−5^	2	From Table A5
Molecular weight (purity)	1	2.80 × 10^−5^ g/mol	0	
Gas constant	2	1.41 × 10^−2^ (cm^2^)/(s^2^K)	0	
RSS	68	7.82 × 10^−8^ g/cm^3^		

**Table A7:** The uncertainty of collection tank gas density for dry air from the NIST Fluid Flow Group small compressor and dryer system

Uncertainty category	Standard uncertainty (*k* = 1)		Contrib	Comments
Collection tank density (air)	Relative (×10^6^)	Absolute	(%)	
Pressure	64	6.45 × 10^−3^ kPa	9	From Table A2
Temperature	22	6.47 mK	1	From Table A4
Compressibility	10	1 × 10^−5^	0	From Table A5
Molecular weight (purity)	190	5.5 × 10^−3^ g/mol	89	
Gas constant	2	1.41 × 10^−2^ (cm^2^)/(s^2^K)	0	
RSS	208	2.38 × 10^−7^ g/cm^3^		

**Table A8:** Collection time uncertainties

Uncertainty category	Standard uncertainty (*k* = 1)		Contrib	Comments
Collection time	Relative (×10^6^)	Absolute (ms)	(%)	
*Time value*		100 000		
Timer cal and usage	0	0.01	0	Base time uncertainty
Inventory correction (start)	2	0.19	50	3000 Hz, rectangular distribution
Inventory correction (stop)	2	0.19	50	3000 Hz, rectangular distribution
RSS	3	0.3		

**Table A9:** Uncertainty of the density of ambient air

Uncertainty category	Standard uncertainty (*k* = 1)		Contrib	Comments
Ambient air density	Relative (×10^6^)	Absolute	(%)	
Pressure	129	0.013 kPa	6	Cals and change during meas
Temperature	336	100 mK	45	Cals and change during meas
Relative humidity	189394	2.5 %	15	Cals and change during meas
Equation of state (*Z*, *M*, *R*)	200	0.0002	33	Based on literature references
RSS	513	6.02 × 10^−7^ g/cm^3^		

**Table A10:** Uncertainty of the external volume of the high pressure cylinder via Archimedes principle

Uncertainty category	Standard uncertainty (*k* = 1)		Contrib	Comments
External tank volume	Relative (×10^6^)	Absolute	(%)	
Apparent mass in air	1	4.61 × 10^−3^ g	0	
Apparent mass in water		0.14 g	34	Based on water *T* uncertainties
Density of air	513	6.01 × 10^−7^ g/cm^3^	0	Correlation and meas unc.
Density of water	20	2 × 10^−5^ g/cm^3^	15	Correlation and meas unc.
Expansion	20	9.38 × 10^−2^ cm^3^	15	*T* and *P* effects
Std deviation of repeated meas.	30	0.14 cm^3^	35	
RSS	51	0.24 cm^3^		

**Table A11:** Uncertainty of the high-pressure cylinder mass measurement

Uncertainty category	Standard uncertainty (*k* = 1)		Contrib	Comments
Final mass of high *P* cylinder	Relative (×10^6^)	Absolute	(%)	
Reference masses	0.5	1.91 × 10^−3^ g	26	From NIST Mass Group cal report
Room air density	513	6.01 × 10^−7^ g/cm^3^	45	
Reference mass density	0	0 g/cm^3^	0	
External cylinder volume	51	0.24 cm^3^	1	From Table 10
Std deviation of repeated meas.	1	0.002 g	28	
RSS	1	0.00376 g		

**Table A12:** Uncertainty of the 677 L collection tank volume

Uncertainty category	Standard uncertainty (*k* = 1)		Contrib	Comments
Collection tank volume	Relative (×10^6^)	Absolute	(%)	
Initial mass of high *P* cylinder	1	0.00385 g	2	See Table A11
Final mass of high *P* cylinder	1	0.00376 g	2	See Table A11
Initial collection tank gas density	100000	1.14 × 10^−9^ g/cm^3^	0	
Final collection tank gas density	68	4.25 × 10^−8^ g/cm^3^	92	See Table A6
Expansion due to *P* and *T*	0	0 cm^3^	0	<1 × 10^−6^
Extra volume uncertainty	1	0.50 cm^3^	0	Related to liq transfer meas.
Std deviation of repeated meas.	16	10.85 cm^3^	5	6 measurements, 2 gases
RSS	71	48.44 cm^3^		

**Table A13:** Uncertainty of the 34 L tank volume determined by the volume expansion method

Uncertainty category	Standard uncertainty (*k* = 1)		Contrib	Comments
Collection tank volume	Relative (×10^6^)	Absolute	(%)	
677 L volume	71	48.4 cm^3^	38	From gravimetric determination
Large inventory volume	1788	1.298 cm^3^	0	From volume expansion method
677 L density change	40	3.53 × 10^−9^ g/cm^3^	12	Dominated by pressure
34 L density change	65	−1.12 × 10^−7^ g/cm^3^	32	Dominated by pressure
Extra volume uncertainty	15	0.5 cm^3^	2	Measured by liq transfer
Std deviation of repeated meas.	46	1.58 cm^3^	16	13 volume determinations
RSS	116	3.95 cm^3^		

**Table A14:** Uncertainties in the inventory pressure measurement, correlated and uncorrelated, for maximum flow conditions

Uncertainty category	Standard uncertainty (*k* = 1)		Contrib	Comments
Inventory pressure measurement	Relative (×10^6^)	Absolute (kPa)	(%)	
*Pressure value*		100		
Sensor calibration	3000	0.30	0	
Sensor time response	1350000	135	100	From inv. model at max flow
Sensed vs stagnation	710	0.071	0	For max. flow
RSS (correlated)	1350004	135		
Sensor repeatability	3.00E+02	0.03	0	From calibration data
Spatial inconsistency	3.00E+04	3	100	Estimated
RSS (uncorrelated)	30001	3.0		

**Table A15:** Inventory temperature uncertainties, correlated and uncorrelated, for the maximum flow for each tank

Uncertainty category	Standard uncertainty (*k* = 1)		Contrib	Comments
Inventory thermocouple	Relative (×10^6^)	Absolute (mK)	(%)	
*Temperature value*		297000		
Sensor calibration	337	100	0	
Sensor time response	168350	50000	97	From inventory model
Sensed vs stagnation	50	15	0	For max. flow
RSS (correlated)	168351	50000		
Sensor repeatability	286	85	0	From calibration data
Spatial inconsistency	30303	9000	100	Estimated
RSS (uncorrelated)	30304	9000		

**Table A16:** Uncertainty of the 1000 cm^3^ (large) inventory volume

Uncertainty category	Standard uncertainty(*k* = 1)		Contrib	Comments
Collection tank volume	Relative (×10^6^)	Absolute	(%)	
677 L volume	71	48.4 cm^3^	0	
677 L density change	1039	3.41 × 10^−9^ g/cm^3^	54	
Inv. vol. density change	120	2.81 × 10^−7^ g/cm^3^	1	
Extra volume uncertainty	526	0.5 cm^3^	14	
Std. deviation of repeated meas.	799	0.76 cm^3^	32	
RSS	1419	1.35 cm^3^		

**Table A17:** Uncertainty of the 75 cm^3^ (small) inventory volume

Uncertainty category	Standard uncertainty (*k* = 1)		Contrib	Comments
Collection tank volume	Relative (×10^6^)	Absolute	(%)	
34 L volume	116	4.0 cm^3^	0	
34 L density change	811	3.41 × 10^−9^ g/cm^3^	24	
Inv. vol. density change	120	2.31 × 10^−7^ g/cm^3^	1	
Extra volume uncertainty	1346	0.1 cm^3^	66	
Std. deviation of repeated meas.	511	0.038 cm^3^	9	
RSS	1661	0.12 cm^3^		

**Table A18:** Uncertainty of the inventory mass change for the 677 L system

Uncertainty category	Standard uncertainty (*k* = 1)		Contrib	Comments
Inventory mass change	Relative (×10^6^)	Absolute	(%)	
Initial density	42643	6.29 × 10^−5^ g/cm^3^	50	3 kPa and 9 K uncorr. inv. unc.
Final density	42643	6.24 × 10^−5^ g/cm^3^	50	3 kPa and 9 K uncorr. inv. unc.
Volume	1419	1.35 cm^3^	0	See Table A16
RSS		8.42 × 10^−2^ g		

**Table A19:** Uncertainty of the inventory mass change for the 34 L system

Uncertainty category	Standard uncertainty (*k* = 1)		Contrib	Comments
Inventory mass change	Relative (×10^6^)	Absolute	(%)	
Initial density	42643	6.29 × 10^−5^ g/cm^3^	50	3 kPa and 9 K uncorr. inv. unc.
Final density	42643	6.24 × 10^−5^ g/cm^3^	50	3 kPa and 9 K uncorr. inv. unc.
Volume	1661	0.123 cm^3^	0	See Table A17
RSS		6.58 × 10^−3^ g		
